# Salinomycin Inhibits Proliferation and Induces Apoptosis of Human Hepatocellular Carcinoma Cells In Vitro and In Vivo

**DOI:** 10.1371/journal.pone.0050638

**Published:** 2012-12-20

**Authors:** Fan Wang, Lei He, Wei-Qi Dai, Ya-Ping Xu, Dong Wu, Chun-Lei Lin, Shu-Mei Wu, Ping Cheng, Yan Zhang, Miao Shen, Chen-Feng Wang, Jie Lu, Ying-Qun Zhou, Xuan-Fu Xu, Ling Xu, Chuan-Yong Guo

**Affiliations:** Department of Gastroenterology, Shanghai tenth People's Hospital, Tongji University of Medicine, Shanghai, People's Republic of China; University of North Carolina School of Medicine, United States of America

## Abstract

The anti-tumor antibiotic salinomycin (Sal) was recently identified as a selective inhibitor of breast cancer stem cells; however, the effect of Sal on hepatocellular carcinoma (HCC) is not clear. This study aimed to determine the anti-tumor efficacy and mechanism of Sal on HCC. HCC cell lines (HepG2, SMMC-7721, and BEL-7402) were treated with Sal. Cell doubling time was determinated by drawing growth curve, cell viability was evaluated using the Cell Counting Kit 8. The fraction of CD133^+^ cell subpopulations was assessed by flow cytometry. We found that Sal inhibits proliferation and decreases PCNA levels as well as the proportion of HCC CD133^+^cell subpopulations in HCC cells. Cell cycle was analyzed using flow cytometry and showed that Sal caused cell cycle arrest of the various HCC cell lines in different phases. Cell apoptosis was evaluated using flow cytometry and Hoechst 33342 staining. Sal induced apoptosis as characterized by an increase in the Bax/Bcl-2 ratio. Several signaling pathways were selected for further mechanistic analyses using real time-PCR and Western blot assays. Compared to control, β-catenin expression is significantly down-regulated upon Sal addition. The Ca^2+^ concentration in HCC cells was examined by flow cytometry and higher Ca^2+^ concentrations were observed in Sal treatment groups. The anti-tumor effect of Sal was further verified *in vivo* using the hepatoma orthotopic tumor model and the data obtained showed that the size of liver tumors in Sal-treated groups decreased compared to controls. Immunohistochemistry and TUNEL staining also demonstrated that Sal inhibits proliferation and induces apoptosis *in vivo*. Finally, the role of Sal on *in vivo* Wnt/β-catenin signaling was evaluated by Western blot and immunohistochemistry. This study demonstrates Sal inhibits proliferation and induces apoptosis of HCC cells *in vitro and in vivo* and one potential mechanism is inhibition of Wnt/β-catenin signaling via increased intracellular Ca^2+^ levels.

## Introduction

Hepatocellular carcinoma (HCC) is the fifth most common cancer worldwide and the third most common cause of cancer deaths, resulting in more than 600,000 deaths annually [1]. Early HCC detection due to advances in diagnostic modalities and clinical screening has made it possible to perform curative or palliative treatment with surgical excision [2]. Unfortunately, HCC is often diagnosed at a late stage, when potentially curative therapies are least effective. For these patients, medical treatments including chemotherapy, chemoembolization, ablation, and proton beam therapy remain disappointing. Most patients show disease recurrence that rapidly progresses to advanced stages with vascular invasion and multiple intrahepatic metastases and the 5-year relative survival rate is only 7%. Furthermore, the prognosis for HCC patients who have surgically resectable, localized tumors remains dismal, with a 5-year recurrence rate of 40–70%. Therefore, there is an urgent need for new therapies for this aggressive disease.

Salinomycin (Sal) is a polyether organic anion used extensively in poultry as a coccidiostatic antibiotic and is commonly fed to ruminant animals to improve feed efficiency [3]. As an ionophore with strict selectivity for alkali ions, it acts in different biological membranes, including cytoplasmic and mitochondrial membranes [4]. Recently, a robotic high-throughput screening approach evaluated approximately 16,000 compounds from chemical libraries for activity against human breast cancer stem cells and found only Sal markedly and selectively reduced the viability of stem-like cells [5]. Additionally, other studies showed Sal could induce apoptosis in chronic lymphocytic leukemia cells [6] and human prostate cancer cells [7]. However, little is known about its impact on HCC cells. HCC is a complex and heterogeneous tumor with several genomic alterations and aberrant activation of several signaling cascades including Wnt, Hedgehog, transforming growth factor-beta (TGF-β), epidermal growth factor receptor (EGFR), vascular epidermal growth factor receptor (VEGFR), mitogen-activated protein kinase (MAPK), and AKT [8].In this study, we aimed to determine the effect of Sal on HCC cells and its underlying mechanisms of action.

## Materials and Methods

### Cell Lines and Culture

The HCC cell lines HepG2, SMMC-7721, and BEL-7402 were purchased from the Chinese Academy of Sciences Committee Type Culture Collection cell bank. The three cell lines were cultured in high glucose Dulbecco's modified Eagle's medium (DMEM-h; Thermo, China) supplemented with 10% fetal bovine serum (Hycione, South America), 100 U/ml penicillin, and 100 µg/ml streptomycin (Gibco,Canada) in a humidified incubator at 37°C in 5% CO_2_.

### Drugs and Antibodies

Sal was purchased from Sigma Aldrich (St. Louis, MO, USA). A 50 mM Sal stock solution was made in dimethyl sulfoxide (DMSO; Gibco, Canada) and stored in the dark at −20°C. The final Sal concentrations used for different experiments were prepared by diluting the stock solution with high-glucose DMEM. The antibodies used for Western blotting and immunohistochemistry staining were as follows: rabbit anti-PCNA, anti-Bcl-2, anti-Bax, anti-p-GSK-3β, anti-GSK-3β, anti-β-catenin, and mouse anti-β-actin. All antibodies were purchased from Santa Cruz Biotechnology (Santa Cruz, CA, USA).

### Cell Growth Curve and Determination of Doubling Time

Three HCC cell lines HepG2, SMMC-7721 and BEL-7402 in logarithmic growth phase were re-suspended, seeded into 24-well plates at a density of 2×10^4^cells/well. One day after seeding, 3 wells were taken to calculate the number of cells as the starting cell number, then Sal was added in three replicates of 0 µM, 2.5 µM, 5 µM, 10 µM for each cell population. Trypan blue staining was performed once daily since the 1st day of culture, and the viable cells in 3 wells of each group were counted for five consecutive days. The doubling time (Td) of each cell line was calculated according to the Patterson formula as follow: Td = T×lg2/(lgN2−lgN1), where N1 is cell number on the 1st day, and N2 is cell number at T h after culture; T (h) is the time from N1 to N2.

### Cell Viability

HepG2, SMMC-7721, and BEL-7402 cells were plated in 96-well plates (100 µl media per well). One day after seeding, Sal was added in five replicates of 0 µM, 5 µM, 10 µM, 15 µM, 20 µM, 25 µM, and 30 µM for each cell population. Cell viability was measured after 24 h, 48 h, and 72 h using the Cell Counting Kit 8 (Peptide Institute Inc., Osaka, Japan) and a microplate reader at a wavelength of 450 nm. A calibration curve was prepared using the data obtained from wells that contained a known number of viable cells.

### CD133^+^ Cell Subpopulation Analyses using Flow Cytometry

HepG2, SMMC-7721, and BEL-7402 cells were plated in 6-well plates. After 48 h, cells from the control group and the Sal [half maximal inhibitory concentration (IC_50_) and 80% inhibitory concentration (IC_80_)] group were collected, washed twice in cold phosphate buffered saline (PBS).Dissociated cells were stained with phycoerythrin-conjugated CD133 antibody (Miltenyi Biotec, Auburn, CA) and incubated for 30 min at 4°C. Mouse IgG1-phycoerythrin was used as isotype control antibody. Dead cells were eliminated with 7-aminoactinomycin D. The labeled cells were analyzed by the BD FACS Vantage Systems in accordance with the manufacturer's protocols. Gating was implemented on the basis of negative-control staining profiles.

### Cell Cycle Analyses using Flow Cytometry

HepG2, SMMC-7721 and BEL-7402 cells were plated in 6-well plates and synchronized with DMEM containing 1% fetal bovine serum. After 48 h, control cells, DMSO-treated cells, and Sal-treated cells (IC_50_) were collected, washed twice in cold PBS, mixed in 300 µl of 1× binding buffer, and incubated at room temperature for 15 min with propidium iodide (PI), NP-40, and RNaseA (BD Biosciences), The cell cycle was analyzed by flow cytometry and the percentage of cells in the different phases was calculated using ModFit LT software (Verity Software House).

### Apoptosis Analyses using Flow Cytometry

HepG2, SMMC-7721 and BEL-7402 cells were plated in 6-well plates. After 48 h, control cells, DMSO-treated cells, and Sal-treated cells (IC_50_) were collected, washed twice in cold PBS, mixed in 100 µl of 1× binding buffer, and incubated at room temperature for 15 min with an annexin-V/PI (BD Biosciences) double staining solution. Stained cells were analyzed by flow cytometry and the percentage of apoptotic cells was calculated using ModFitLT software (Verity Software House).

### Hoechst 33342 Staining

HepG2, SMMC-7721 and BEL-7402 cells were plated in 24-well plates. After 48 h, control cells, DMSO-treated cells, and Sal-treated cells (IC_50_) were washed twice in PBS. Hoechst 33342 staining (Sigma Aldrich) solution (1 µl added to 200 µl PBS) was added to each well for 20–30 minutes at 4°C in the dark. Blue fluorescent cells were examined using fluorescence microscopy.

### Intracellular Calcium Analyses using Flow Cytometry

HepG2, SMMC-7721 and BEL-7402 cells were plated in 6-well plates. After 48 h, control cells, DMSO-treated cells, and Sal-treated cells (IC_50_) were collected, washed twice in cold PBS, mixed in 300 µl 1× binding buffer, and incubated at room temperature for 30 min with intracellular calcium fluorescent probes (fluo-3) (Sigma Aldrich) staining solution. The intracellular calcium levels were analyzed by flow cytometry and the mean fluorescence intensity was calculated using ModFit LT software (Verity Software House).

### Animal Experiments

Animal experiments were performed on 6-week-old male nude mice (athymic, BALB/C nu/nu). Mice were housed in a standard animal laboratory with free access to water and food. They were kept under constant environmental conditions with a 12-hour light-dark cycle. All operations were performed under aseptic conditions. All procedures were approved by the Animal Care and Use Committee of Shanghai Tongji University.

### Hepatoma Orthotopic Tumor Model and Treatment Regimen

Nude mice (nu/nu; 4–6 weeks of age) were purchased from Shanghai Slac Laboratory Animal Co., Ltd. HepG2 cells were suspended in 100 ml1∶1 serum-free DMEM and Matrigel (BD Biosciences). Mice were anesthetized with ketamine/xylazine and after surgically opening the abdomen, HepG2 cells were inoculated into the liver parenchyma and mice were monitored every 3 days for 35 days. Finally, 18 nude mice were divided into three groups that were intraperitoneally injected daily for 6 weeks: two Sal-treated groups (4 mg/kg Sal group, 8 mg/kg Sal group) and the control group (saline water group)

### Immunohistochemistry

Tumor tissue from control group (saline water group) and Sal-treated groups are used for immunohistochemistry. Sections (4 µm thick) from paraffin-embedded tumors were deparaffinized and rehydrated using xylene and ethanol, respectively, and immersed in 3% hydrogen peroxide solution for 10 min to block endogenous peroxidases. Sections were boiled for 30 min in 10 mM citrate buffer solution (pH 6.0) for antigen retrieval. Slides were incubated for 45 min with 5% bovine serum albumin and incubated overnight at 4°C with anti-PCNA, anti-Bcl-2, anti-Bax, or anti-β-catenin. These specimens were incubated for 45 min at 37°C with the appropriate peroxidase-conjugated secondary antibody and visualized using the Real Envision Detection Kit (Gene Tech Shanghai Company Limited, China) following the manufacturer's instructions. All slides were counterstained with hematoxylin and eosin (HE).

### Terminal Deoxynucleotidyl Transferase dUTP Nick End Labeling (TUNEL) assays

Tumor tissue from control group (saline water group) and Sal-treated groups are used for TUNEL assays. Sections (4 µm) from formalin fixed, paraffin-embedded tumors were deparaffinized and rehydrated using xylene and ethanol, respectively. The slides were rinsed twice with PBS and treated for 15 min at 37°C with proteinase K (15 µg/ml in 10 mM Tris/HCl, pH 7.4–8.0). Endogenous peroxidases were blocked using 3% hydrogen peroxide in methanol at room temperature for 10 min. The tissue sections were then analyzed with an *in situ* Cell Death Detection Kit-POD (Roche, Germany) in accordance with the manufacturer's instructions. The reaction was visualized using fluorescence microscopy.

### Reverse Transcription-Polymerase Chain Reaction (RT- PCR) and Real-Time PCR

Total RNA was extracted and first-strand cDNA was synthesized using the Omniscript RT kit (Qiagen), with 2000 ng of RNA (per 20-µl reaction) and oligo (dT) primers. cDNA was then utilized in real-time PCR reactions to analyze PCNA, Bcl-2, Bax, DKK1, DKK2, GSK-3α, GSK-3β, β-catenin, Gli-1, Patched, Shh, Smo, TGF-β, Smad1, Smad2, Smad3, Smad4, Smad5, Smad8, EGFR, VEGFR, MAPK, AKT, and β-actin expression. Primers used in the PCR reactions are presented in Table 1. PCR reactions were amplified for 40 cycles. Each cycle consisted of denaturation for 1 min at 94°C, annealing for 1 min at 60°C, and polymerization for 2 min at 72°C. PCR products were quantified using the Molecular Analyst software (Bio-Rad). The ratio of each gene against β-actin was calculated by standardizing the ratio of each control to the unit value.

**Table 1:** Real-time PCR Primer Sequences.

Gene		Primer sequence (5′→3′)	Gene		Primer sequence (5′→3′)
PCNA	Forward	GCTGACATCGGACACTTA	TGF-β	Forward	GAAACCCACAACGAAATCTA
	Reverse	CTCAGGTACAAACTTGGTG		Reverse	GGCGAAAGCCCTCAAT
Bcl-2	Forward	CATGTGTGTGGAGAGCGTCAA	Smad1	Forward	CAACAGCCACCCGTTTCCT
	Reverse	GCCGGTTCAGGTACTCAGTCA		Reverse	GTTTGGGTAACTGCTATTGGGA
Bax	Forward	GATCCAGGATCGAGCAGA	Smad2	Forward	TCATAGCTTGGATTTACAGCCAG
	Reverse	AAGTAGAAGAGGGCAACCAC		Reverse	ACACCAAAATGCAGGTTCTGAG
DKK1	Forward	ATTCCAGCGTTGTTACTGTGG	Smad3	Forward	TGGACGCAGGTTCTCCAAAC
	Reverse	GATCTTTCTGTATCCGGCAAGAC		Reverse	GTGCTGGGGACATCGGATTC
DKK2	Forward	GATCTGCGGGCATGTACCAA	Smad4	Forward	CTCATGTGATCTATGCCCGTC
	Reverse	TCCTTATCACTGCTACAAGGGT		Reverse	AGGTGATACAACTCGTTCGTAGT
GSK-3α	Forward	AGTGGCTTACACGGACATCCA	Smad5	Forward	CCAGCAGTAAAGCGATTGTTGG
	Reverse	CTGGTACACGACCCCAAATGA		Reverse	GGGGTAAGCCTTTTCTGTGAG
GSK-3β	Forward	AGACGCTCCCTGTGATTTATGT	Smad8	Forward	ATGTGATTTACTGCTGCGTGT
	Reverse	CCGATGGCAGATTCCAAAGG		Reverse	CGGTAGTGGTAAGGGTTAATGC
β-catenin	Forward	TACCGTTGGATTGATTCG	EGFR	Forward	GGACGACGTGGTGGATGC
	Reverse	GTCAGAGGTGCTGTGGCT		Reverse	GGCGCCTGTGGGGTCTGAGC
Gli-1	Forward	TCTGCCCCCATTGCCCACTTG	VEGFR-1	Forward	CTGACTCTCGGACCCCTG
	Reverse	TACATAGCCCCCAGCCCATACCTC		Reverse	TGGTGCATGGTCCTGTTG
Patched	Forward	CGGCGTTCTCAATGGGCTGGTTTT	MAPK	Forward	CCGCCTCAGTATGCAGTCCA
	Reverse	GTGGGGCTGCTGTTTCGGGTTCG		Reverse	GCCACATGTGCAAAGGCATC
Shh	Forward	CGGAGCGAGGAAGGGAAAG	AKT	Forward	ATGAGCGACGTGGCTATTGTGAAG
	Reverse	TTGGGGATAAACTGCTTGTAGGC		Reverse	GAGGCCGTCAGCCACTCTGGATG
Smo	Forward	ACCCCGGGCTGCTGAGTGAGAAG	β-actin	Forward	CTGGAACGGTGAAGGTGACA
	Reverse	TGGGCCCAGGCAGAGGAGACATC		Reverse	AAGGGACTTCCTGTAACAATGCA

### Western Blot Assays

HepG2, SMMC-7721 and BEL-7402 cells were treated with Sal (IC50) for two days. Cells were washed twice with PBS solution, then cells were lysed with RIPA Lysis Buffer (Beyotime Institune of Biotechnology, Shanghai, China) and protease inhibitor (Thermo Scientific). Protein concentrations were determined with Pierce BCA Protein Assay Kit (Thermo Scientific), Equivalent amounts of total protein (60 ug) were boiled and electrophoretically separated on a 10% polyacrylamide gel at 80 volts. The proteins were transferred to a polyvinylidene difluoride membrane. Membranes were blocked for 60 min with a 5% milk solution prepared in PBS. The membranes were incubated overnight at 4°C with 1∶500 dilutions of the primary antibodies (PCNA, Bcl-2, Bax, p-GSK-3β, GSK-3β, β-catenin, and β-actin). Membranes were washed three times for 5 min each with Tween 20(1∶1000 dilution)-PBS and incubated for 45 min with the appropriate peroxidase-conjugated secondary antibody (1∶1000 dilution). Membranes were washed with Tween 20-PBS three times for 10 min each and were developed using the Odyssey two-color infrared laser imaging system. The signal generated by β-actin was used as an internal control.

### Statistical Analyses

The SPSS17.0 software was used for statistical analyses. Experiments were repeated at least three times with consistent results. Unless otherwise stated, data are expressed as the mean ± standard deviation. The data of Real-time PCR were 2-ΔΔCt transformed before analysis and were analyzed using ANOVA, the results of CCK8 assay were analyzed by two-Way Analysis of Variance, The results of Western blots, CD133, cell cycle, annexin-V/PI, and fluo-3 staining were analyzed using analysis of ANOVA. Tumor mean diameter, and mean volume were analyzed for statistical significance using paired Student's t tests. In all cases, *p*<0.05 was considered significantly statistic difference.

## Results

### Sal Inhibits HCC Cell Proliferation in vitro

**Figure 1:**
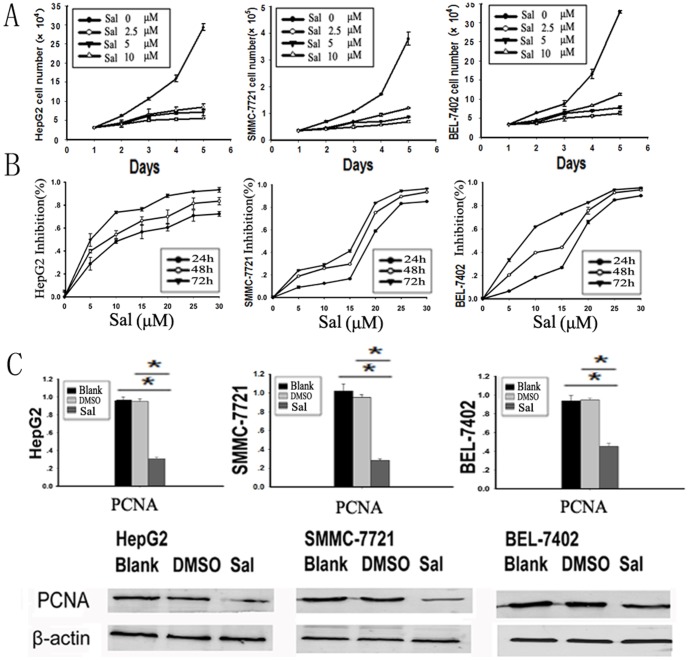
Sal inhibits HCC cell proliferation *in vitro*. A. Growth curve of HCC cells. HepG2, SMMC-7721, and BEL-7402 cells were treated with Sal (0, 2.5, 5, 10 µM) for five days. The growth of Sal-treated cells was relatively slower than that of DMSO-treated cells in a dose-dependent manner. B. Sal inhibits HCC cell proliferation. HepG2, SMMC-7721, and BEL-7402 cells (5×10^4^ cells/ml) were treated with Sal (0–25 µM) at various time points (24, 36, and 48 h). Cell viability was determined using a CCK8 assay. The data show that Sal exposure reduced HepG2, SMMC-7721, and BEL-7402 cells in a dose- and time-dependent manner. C. Sal down-regulates the mRNA expression (*p<0.005) and protein expression of PCNA in HepG2, SMMC-7721, and BEL-7402 cells.

The HCC cell lines HepG2, SMMC-7721 and BEL-7402 were treated with increasing concentrations of Sal for five days and we drew the growth curve (Fig. 1A). The data showed that the growth of Sal-treated cells was relatively slower than that of DMSO-treated cells in a dose-dependent manner, the Td of Sal-treated HepG2, SMMC-7721, and BEL-7402 cells were significantly longer than that of DMSO-treated cells (Table 2,* *p*<0.05). Sal cytotoxicity was determined using a CCK8 assay. The data indicate that Sal reduced the viability of HepG2, SMMC-7721, and BEL-7402 cells in a dose- and time-dependent manner (Fig. 1B; *p*<0.05). The Sal concentrations that reduced growth by 50% and 80% (IC_50_ and IC_80_) are summarized in Table 3. We evaluated PCNA expression by real-time PCR and Western blot. The results showed that Sal could down-regulate the expression of PCNA mRNA and protein (Fig. 1C; * *p*<0.05)..

**Table 2:** Time doubling of HCC cells.

Time doubling (h)			
	HepG2	SMMC-7721	BEL-7402
DMSO	29.6	28.2	29.2
Sal 2.5 µM	66.6*	54.9*	55.1*
Sal 5 µM	80.8*	74.7*	78.3*
Sal 10 µM	116.1*	101.4*	105.6*

* P<0.05 were significantly statistic difference between Sal groups and DMSO groups.

**Table 3:** Growth-inhibitory activity of Sal on HCC cells.

Sal (µM)	HepG2		SMMC-7721		BEL-7402	
	IC_50_	IC_80_	IC_50_	IC_80_	IC_50_	IC_80_
24 h	14.7	26.2	18.6	27.8	17.1	25.7
48 h	10.2	18.5	15.3	23.8	13.8	22.2
72 h	7.7	14.7	13.7	21.6	10.4	18.8

### Sal Reduces the Proportion of HCC CD133^+^ Cell Subpopulations in vitro

**Figure 2:**
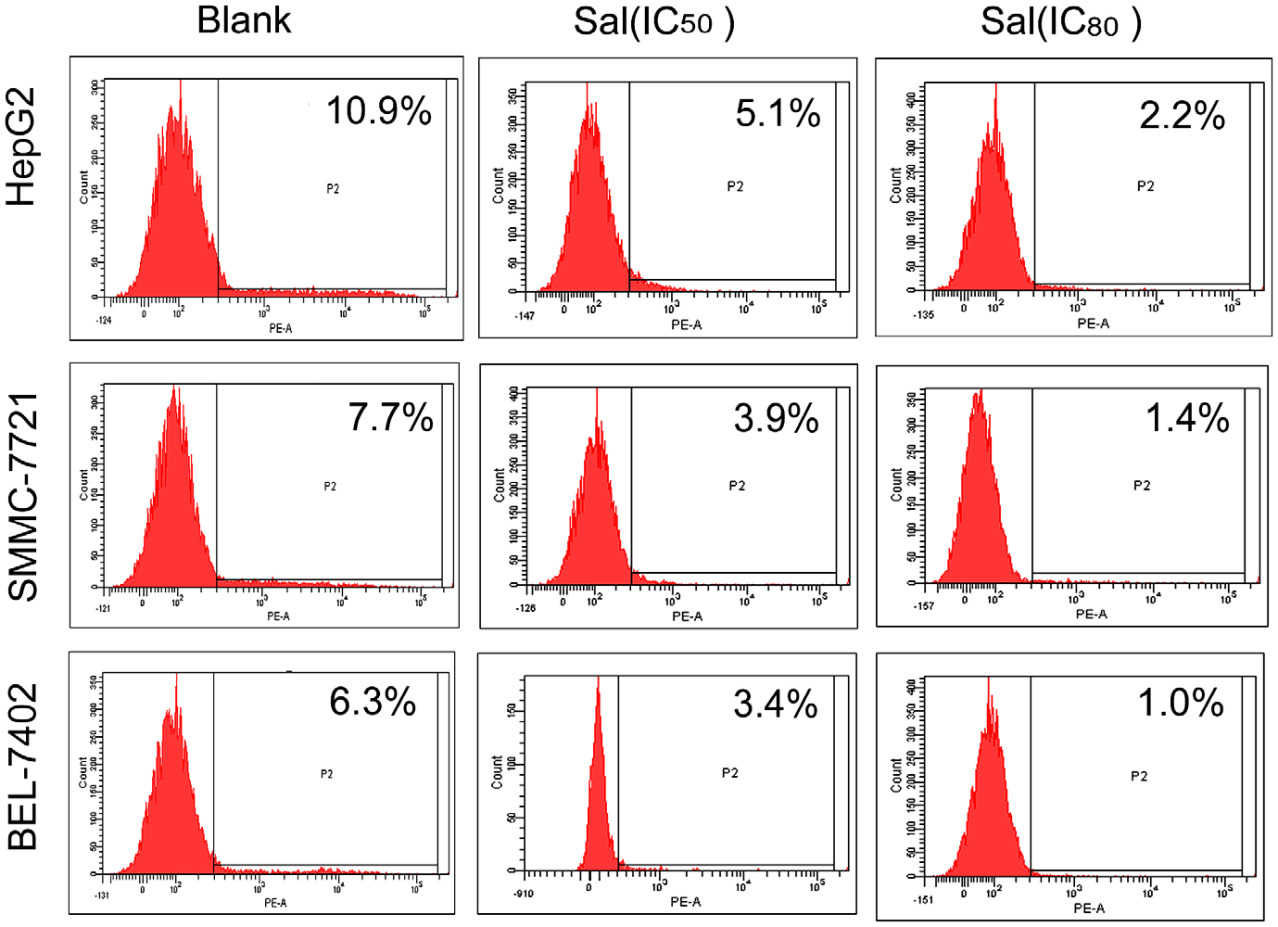
Sal reduces the proportion of HCC CD133^+^ cell subpopulations *in vitro*. Sal (IC_50_ and IC_80_) reduces the proportion of HCC CD133+ cell subpopulations in a dose-dependent manner.

A small population of cancer cells, termed cancer stem cells, have unlimited proliferation, self-renewal, and multi-lineage differentiation potentials. The CD133+ protein, a pentaspan cell surface receptor, is a putative HCC stem cell marker. Flow cytometry was performed to analyze the effect of Sal on the proportion of HCC cells with a CD133+ antigenic phenotype. Sal reduced the proportion of CD133+ cells in HepG2 cells (Blank 10.9%; Sal-IC50 5.1%; Sal-IC80 2.2%), SMMC-7721 cells (Blank 7.7%; Sal-IC50 3.9%; Sal-IC80 1.4%), and BEL-7402 cells (Blank 6.3%; Sal-IC50 3.4%; Sal-IC80 1.0%) in a dose -dependent fashion (Fig. 2; p<0.05).

### Sal Causes Cell Cycle Arrest of HCC Cells in vitro

**Figure 3:**
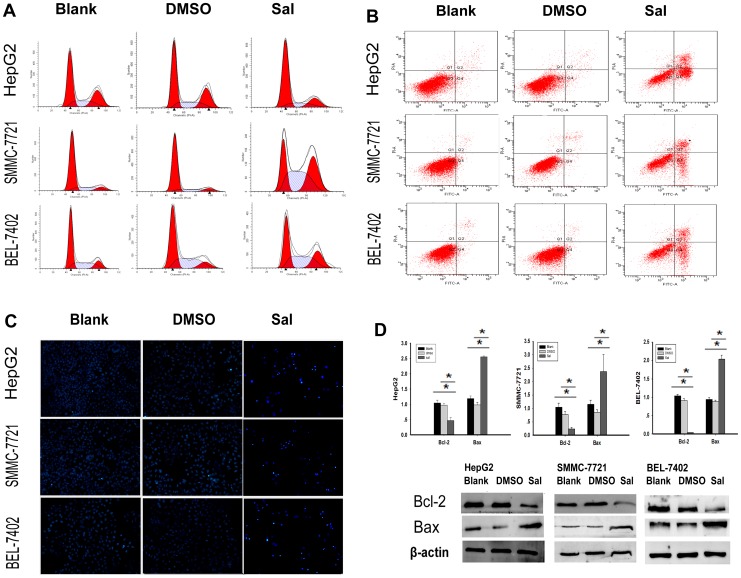
Sal causes cell cycle arrest and induces apoptosis of HCC cells *in vitro*. A. Sal causes cell cycle arrest of different HCC cells in different phases of the cell cycle. Sal treatment (10.2 µM) for 48 h in HepG2 cells caused cell cycle arrest at the G0/G1 phase, whereas treatment with 15.3 µM Sal and 13.8 µM Sal for 48 h in SMMC-7721 and BEL-7402 cells, respectively, induced G2/M phase arrest. B. Sal induces HCC cell apoptosis.Flow cytometric analyses of annexin-V/PI staining of HepG2, SMMC-7721, and BEL-7402 cells. Cells were treated with Sal (IC_50_) for 48 h. The dual parameter dot plots combining annexin-V-fluorescein isothiocyanate (FITC) and PI fluorescence show the viable cell population in the lower left quadrant (annexin-V−, PI−), apoptotic cells in the lower right quadrant (annexin-V+, PI−) and upper right quadrant (annexin V+, PI+),and necrotic cells in the upper left quadrant (annexin-V−, PI+). C. Morphological changes of HepG2, SMMC-7721, and BEL-7402 cells. After treatment with Sal (IC_50_) for 48 h, nuclear fragmentation was observed using laser scanning microscopy. Magnification 200×. D. Real-time PCR analyses and Western blotting revealed that Sal treatment of HepG2, SMMC-7721, and BEL-7402 cells increased the Bax/Bcl-2 ratio (*p<0.05).

In this study, we found that Sal could inhibit proliferation of the HCC cell lines. Therefore, we investigated the mechanism of this inhibition. The effect of Sal on cell cycle was tested using flow cytometry analyses. As shown in Figure 3A and Table 4 (*p<0.05), treatment of 10.2 µM Sal for 48 h caused G0/G1 cell cycle arrest in HepG2 cells, whereas 15.3 µM Sal and 13.8 µM Sal for 48 h induced G2/M phase arrest in SMMC-7721 and BEL-7402 cells, respectively. Our results reveal that Sal triggers different cell phase arrest in different HCC cells.

**Table 4:** Cell cycle analyses of HCC cells.

	HepG2			SMMC-7721			BEL-7402		
	G0/G1	G2/M	S	G0/G1	G2/M	S	G0/G1	G2/M	S
Blank group	56.3±4.9	25.0±2.3	18.7±2.4	68.0±4.8	10.1±2.0	18.6±4.2	53.0±2.6	15.0±2.2	32.0±1.5
DMSO group	52.0±1.9	27.8±1.1	20.2±2.9	78.2±1.9	9.1±1.6	12.7±3.4	51.1±2.5	12.0±1.4	36.9±1.1
Sal group	66.8±4.0*	15.8±1.2	17.4±3.9	25.4±2.0	32.7±1.8*	41.9±3.8	36.9±2.3	23.6±1.5*	39.4±3.7

* P<0.05 were significantly statistic difference between Sal groups and Blank groups, DMSO groups.

### Sal Induces HCC cell Apoptosis in vitro

To examine if Sal could induce apoptosis, we used flow cytometry analyses (Fig. 3B and Table 5, * *p*<0.05). An increase in the percentage of early apoptosis (quadrant 2) plus late apoptosis (quadrant 3) was identified in HepG2, SMMC-7721, and BEL-7402 cells after Sal treatment. Cell morphology changes were also observed using Hoechst 33342 staining (Fig. 3C). Using a laser-scanning microscope, cell nuclei were round and homogeneous in the absence of Sal, whereas Sal treatment resulted in nuclear condensation, a hallmark feature of apoptotic cells. Moreover, real-time PCR and Western blotting revealed that Sal treatment increased the Bax/Bcl-2 ratio in HepG2, SMMC-7721, and BEL-7402 cells (Fig. 3D, * *p*<0.05).

**Table 5:** Apoptosis analyses of HCC cells.

	Apoptosis rate (%)		
	HepG2	SMMC-7721	BEL-7402
Blank group	5.1±0.9	5.0±0.8	5.2±1.0
DMSO group	4.5±0.2	4.3±0.8	4.4±0.6
Sal group	30.0±4.7*	26.6±1.0*	30.3±3.5*

* P<0.05 were significantly statistic difference between Sal groups and Blank groups, DMSO groups.

### Sal Represses the Wnt/β-catenin Signaling Pathway in vitro

**Figure 4:**
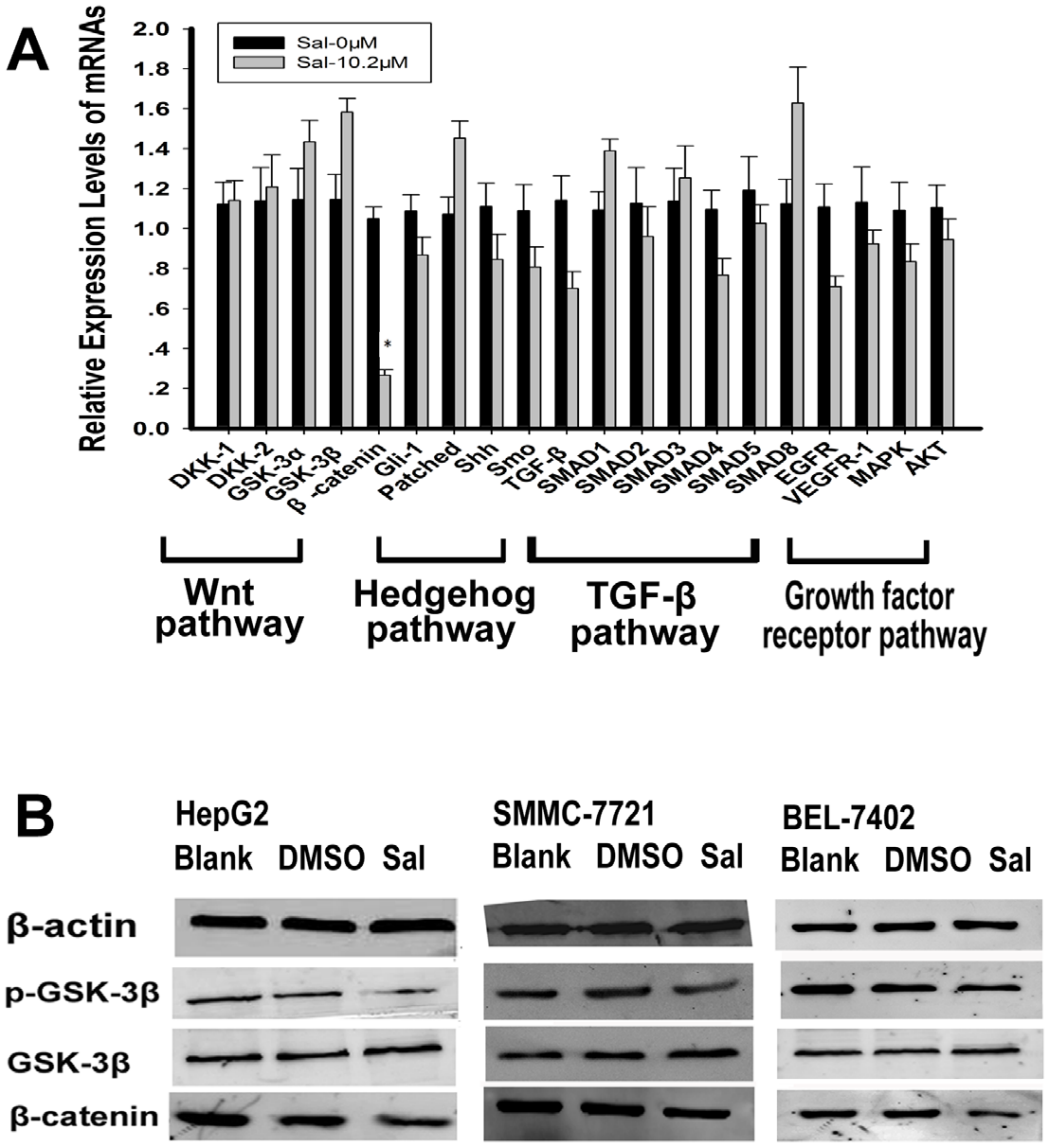
Sal represses the Wnt/β-catenin signaling pathway and increases intracellular calcium *in vitro*. A. Real-time PCR was performed to examine mRNA expression of the Wnt pathway (DKK1, DKK2, GSK-3α, GSK-3β, and β-catenin), the hedgehog pathway (Gli-1, Patched, Shh, and Smo), the TGF-β pathway (TGF-β, Smad1, Smad2, Smad3, Smad4, Smad5, and Smad8), and growth factor receptor pathways (EGFR, VEGFR, AKT, and MAPK). Compared to control, the expression of β-catenin is significantly down-regulated (*p<0.05). B. Sal inhibits Wnt/β-catenin signaling in HepG2, SMMC-7721, and BEL-7402 cells. p-GSK-3β, GSK-3β, and β-catenin expression were evaluated by Western blot.

Next, we examined the Wnt pathway (including DKK1, DKK2, GSK-3α, GSK-3β, and β-catenin), the hedgehog pathway (including Gli-1, Patched, Shh, and Smo), the TGF-β pathway (including the TGF-β, Smad1, Smad2, Smad3, Smad4, Smad5, and Smad8), and growth factor receptor pathways (including EGFR, VEGFR, AKT, and MAPK) via real-time PCR. Compared to controls, β-catenin expression is significantly down-regulated (Fig. 4A; * *p*<0.05). We examined protein expression of β-catenin by Western blot and found a similar change in β-catenin protein. Decreased p-GSK-3β protein was also observed, indicating GSK3β activity, the upstream regulatory factor of β-catenin, was activated by Sal. Thus, Sal plays an inhibitory role in the regulation of Wnt/β-catenin signaling pathway (Fig. 4B; *p*<0.05).

### Sal Increases Intracellular Calcium Levels in vitro

**Figure 5:**
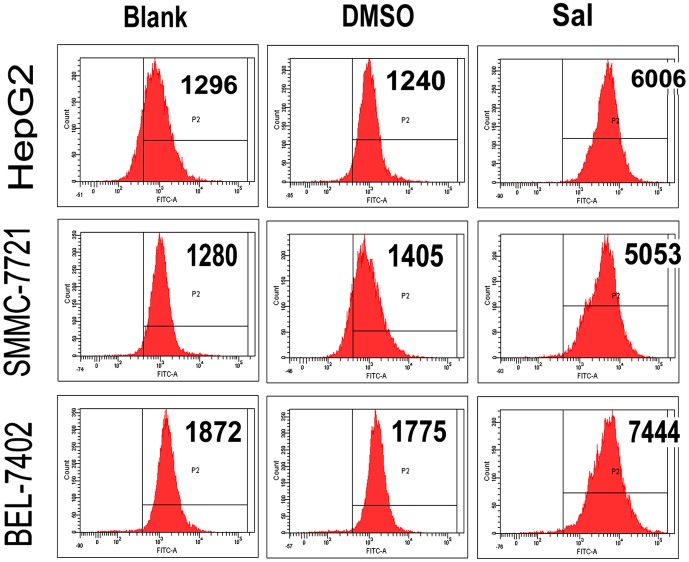
Sal increases intracellular Calcium levels *in vitro*. Sal elevates intracellular calcium levels. The mean intracellular calcium levels of HepG2, SMMC-7721 and BEL-7402 cells were noticeably elevated after Sal treatment.

To assess whether Sal elevates intracellular calcium levels, Fluo-3 FACS analyses were used. As shown in Figure 3C, the mean fluorescence intensity of intracellular calcium was remarkably increased in HepG2, SMMC-7721, and BEL-7402 cells after treatment with Sal (IC_50_; Fig. 5).

### Anti-tumor Activity of Sal in vivo

**Figure 6:**
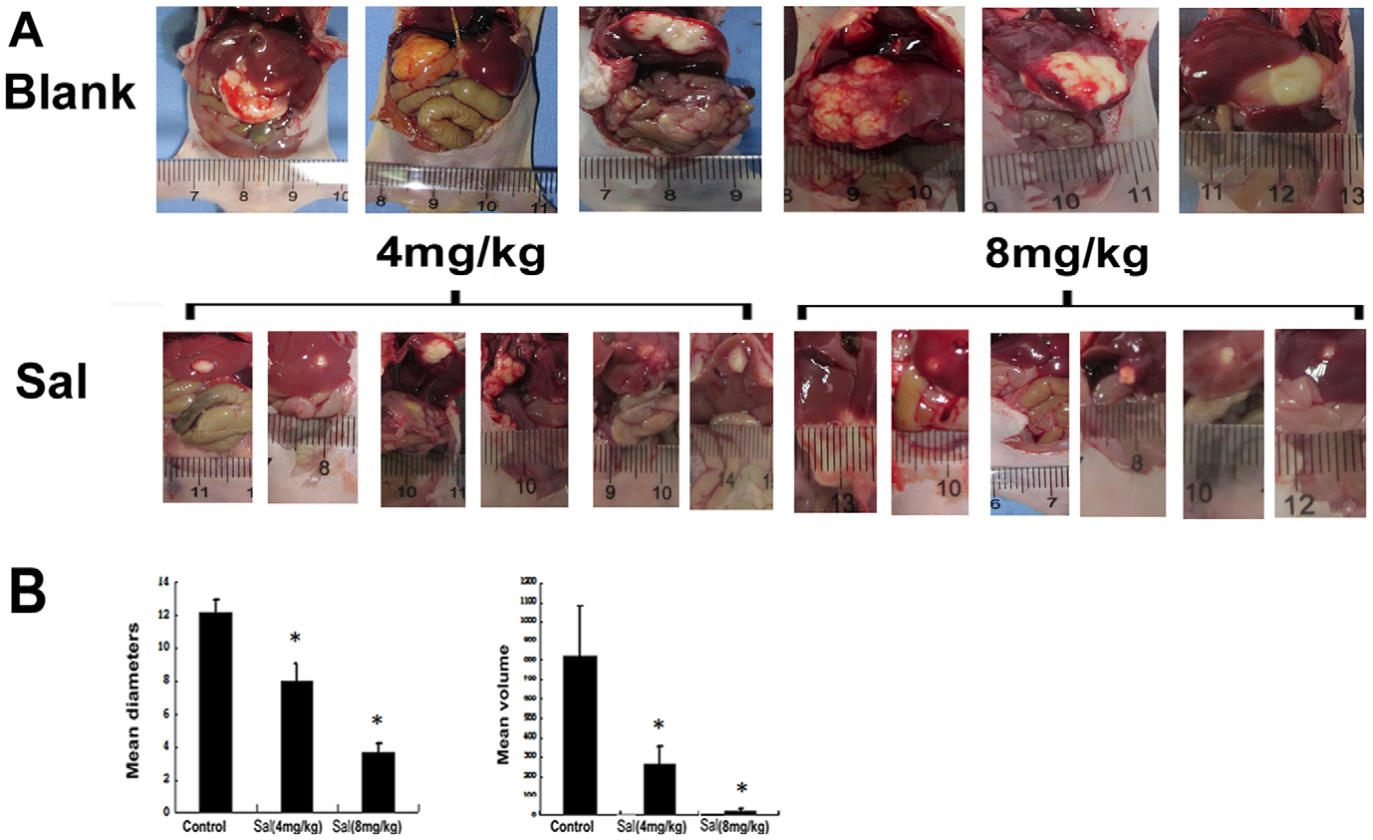
Sal reduce the growth of HepG2 orthotopic tumor *in vivo*. A. Gross observation of HepG2 cell orthotopic tumors in nude mice from the saline group or Sal groups (4 mg/kg or 8 mg/kg). B. Tendency of tumor mean diameter after injection in nude mice (*p<0.05). Tendency of tumor mean volume after injection in nude mice (*p<0.05).

**Figure 7:**
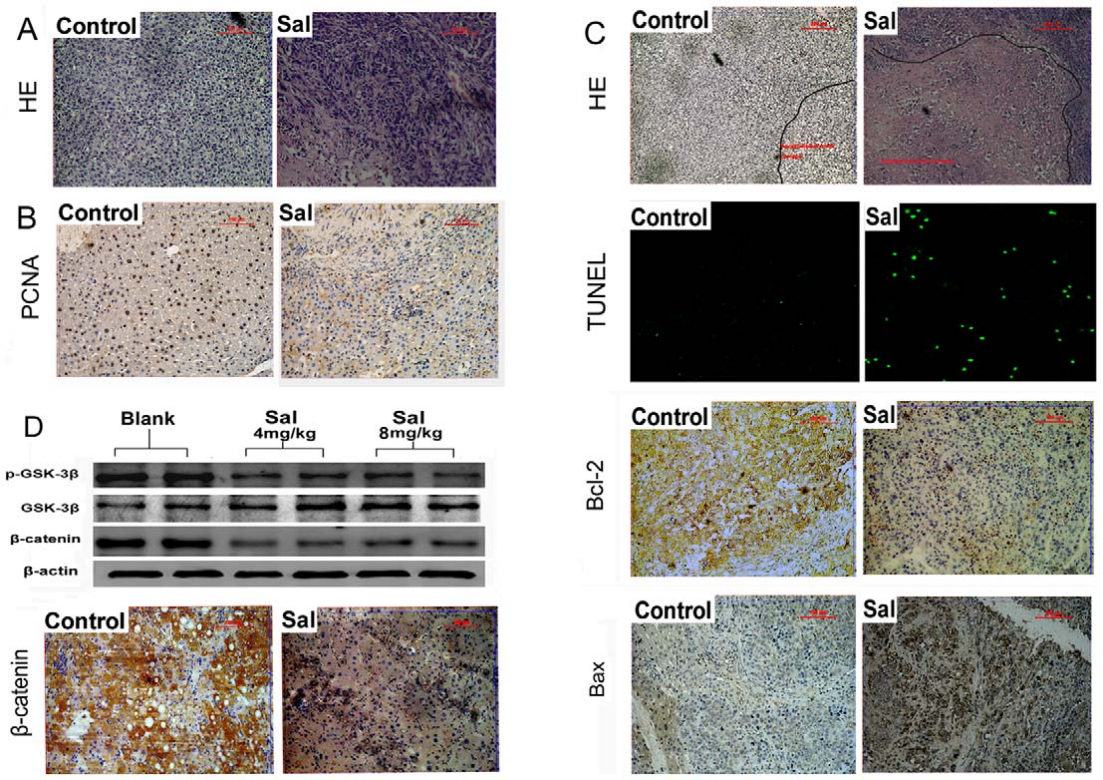
Anti-tumor activity of Sal *in vivo*. A. HE staining showed the structure of the liver cancer tissue: nuclei of different sizes, hepatic cord structure was destroyed, Magnification 200×. B. Immunohistochemistry indicates that PCNA expression is down-regulated after Sal treatment. Magnification 200×. C.HE staining showed apoptotic and necrotic areas(nucleus disintegration, organizational integration) increased after Sal treatment. Magnification 100×. TUNEL apoptotic assay indicates increased apoptosis in Sal-treated tumor tissue. Immunohistochemistry shows that Bcl-2 expression was down-regulated and Bax expression was up-regulated after Sal treatment. Magnification 200×. D. Sal inhibits Wnt/β-catenin signaling pathways. Expression of p-GSK-3β, GSK-3β, and β-catenin was evaluated by Western blot. Immunohistochemistry showed that β-catenin expression was down-regulated after Sal treatment. Magnification 200×.

In order to investigate the effect of Sal on anti-tumor activities *in vivo*, we established a HepG2 orthotopic tumor model in nude mice. After administration of 4 mg/kg Sal, 8 mg/kg Sal and 10 ul/g saline water for 6 weeks, the mice were sacrificed. The size of the liver tumors in the Sal treatment groups diminished compared with the control group (Fig. 6A). The mean diameter of the tumors decreased from 12.17 mm to 3.67 mm (Fig. 4B, * *p*<0.05) and the mean volume (V = length×width2×0.5) [9] of the tumors decreased from 819 mm^3^ to 25.25 mm^3^ (Fig. 6B, * *p*<0.05). Next, the tumors were harvested, followed by HE staining, immunohistochemistry, and TUNEL assays, to assess the anti-tumor activity of Sal *in vivo*. HE staining (Fig. 7A) showed that the structure of the liver cancer tissue∶nuclei of different sizes, hepatic cord structure was destroyed. Immunohistochemistry showed that PCNA expression (Fig. 7B) was lower after Sal treatment. HE staining (Fig. 7C) and TUNEL (Fig. 7C) assays indicated the Sal-treated groups had higher apoptosis rates than control. Furthermore, immunohistochemistry showed an increased Bax/Bcl-2 ratio after Sal treatment (Fig. 7C). Finally, we examined *in vivo* expression of p-GSK-3β, total GSK-3β, and β-catenin using Western blotting. Expression of p-GSK-3β decreased but total GSK-3β did not change, indicating increased GSK3β activity. The protein expression of β-catenin decreased in the Sal treatment groups compared with control (Fig. 7D). Finally, we evaluated expression of β-catenin using immunohistochemistry (Fig. 7D) and verified Sal could down-regulate the β-catenin expression in HCC.

## Discussion

It has been reported that Sal can decrease the malignant traits of several types of cancer cells [10], [7]. In this study, we found that Sal inhibited proliferation of HCC cell lines and reduced the proportion of HCC CD133+ cell subpopulations. Recent studies support the theory that tumors can be initiated and maintained by a small population of cells that have stem-like features, and these highly tumorigenic cells within the tumor bulk are considered cancer stem cells [11]. Studies have identified CD133 as an adequate marker of HCC cancer stem cells [12]–[14]. CD133-expressing cancer cells with a stem-cell-like phenotype are believed to account for chemotherapy resistance, tumor recurrence, and disease progression. Compared with paclitaxel [5] and oxaliplatin [15] that kill general cancer cells, Sal selectively kills cancer stem cells, providing a new strategy for cancer therapy.

In this study, we show that Sal repressed HCC cell proliferation. Cell-cycle dysregulation and apoptosis resistance are hallmarks of tumor cells. Sal caused different cell phase arrests in different HCC cells; however, our data showed the induction of apoptosis in Sal-treated HCC cells was similar. Apoptosis resistance is one of the main causes of tumorigenesis and tumor drug resistance [16]. The Bcl-2 family plays a critical role in apoptosis regulation. Pro-apoptotic Bax promotes intrinsic apoptosis by forming oligomers in the mitochondrial outer membrane, facilitating the release of the apoptogenic molecules, whereas anti-apoptotic Bcl-2 blocks mitochondrial apoptosis by blocking the release and oligomerization of Bax [17]. Sal can induce apoptosis and overcome apoptosis resistance of hepatoma cells *in vitro and in vivo*, which might make it an effective chemotherapy agent for treating HCC.

β-catenin, encoded by the CTNNB1 gene, has multiple functions, including mediation of cell adhesion and signal transduction. It combines with a variety of proteins to regulate cell proliferation and differentiation, which is critical for embryonic development and tumorigenesis. In agreement with another report [18], this study demonstrates that Sal inhibited proliferation and promoted hepatoma apoptosis *in vitro and in vivo* through down-regulating β-catenin expression. The β-catenin gene mutation rate is approximately 20% in HCC [19], [20]. However, β-catenin accumulated within 67% of HCC tissues and is closely related to the clinicopathological features of HCC [21]–[23]. It is likely that some upstream molecules play an important role in β-catenin activation in HCC. In canonical Wnt signaling pathways, Gsk-3β is the upstream adjustment factor of β-catenin and can compose a complex with APC and axin to phosphorylation β-catenin, leading to β-catenin degradation. Gsk-3β is inactivated by phosphorylation as well because in this study, we showed that Sal suppressed GSK-3β phosphorylation, suggesting active GSK-3β was up-regulated, followed by β-catenin degradation.

Sal functions on different biological membranes, including cytoplasmic and mitochondrial membranes, as an ionophore with strict selectivity for alkali ions and a strong preference for potassium and it can disrupt Na^+^/Ca^2+^ exchange and cause an increase in intracellular calcium levels [24]. Previous studies have demonstrated that non-canonical Wnt family members repress canonical Wnt signaling by inducing calcium influx and the increase in intracellular calcium inhibits the canonical Wnt pathway [25]–[29]. Here, we show that Sal can increase intracellular calcium levels and inhibit the Wnt pathway. Moreover, increased intracellular Ca^2+^ is an initiating factor of apoptosis because nucleosome DNA degradation is calcium-dependent, providing a reasonable explanation of Sal-induced apoptosis [30].

In this study, we demonstrate the anti-tumor antibiotic Sal can inhibit proliferation, induce apoptosis, and repress canonical Wnt/β-catenin signaling involved in HCC growth via increasing intracellular calcium levels. Although the exact mechanism remains unclear, this research provides a starting point for exploring Sal-related HCC cancer therapy.
